# The Concentration of Salivary Extracellular Vesicles Is Related to Obesity

**DOI:** 10.3390/nu16162633

**Published:** 2024-08-09

**Authors:** Kristin Röhrborn, Martin Krueger, Mirjam Kalusa, Simone A. Fietz, Alexander Ewe, Achim Aigner, Michael Stumvoll, Peter Kovacs, Matthias Blüher, Imke Schamarek, Kerstin Rohde-Zimmermann

**Affiliations:** 1 Helmholtz-Institute for Metabolic, Obesity and Vascular Research (HI-MAG), Helmholtz Center Munich at the University of Leipzig and the University Hospital Leipzig AöR, 04103 Leipzig, Germany; kristin.roehrborn@helmholtz-munich.de (K.R.);; 2 Institute of Anatomy, Medical Faculty, University of Leipzig, 04103 Leipzig, Germany; 3 Institute of Veterinary Anatomy, Histology and Embryology, Faculty of Veterinary Medicine, University of Leipzig, 04103 Leipzig, Germany; 4 Rudolf-Boehm Institute for Pharmacology and Toxicology, Clinical Pharmacology, University of Leipzig, 04103 Leipzig, Germany; 5 Department of Medicine III, Division of Endocrinology, Nephrology and Rheumatology, University of Leipzig, 04103 Leipzig, Germany

**Keywords:** saliva, extracellular vesicles, obesity, taste bud

## Abstract

Background and aims: Saliva is essential for the proper dilution and distribution of taste molecules on the tongue. It harbors extracellular vesicles (EVs), which mediate cell–cell communication. Changes in the composition of salivary EVs may arise under obese conditions and may potentially be involved in taste sensation and dysregulated eating behavior. Therefore, this study addresses the relationship between the size and concentration of salivary EVs and metabolic shifts in obesity or factors of taste sensation. Materials and methods: A total of 119 participants in the Obese Taste Bud (OTB) Study were included, who performed a standardized taste test, underwent taste bud density assessment, and were phenotypically characterized for anthropometrics, blood- and saliva adipokine levels, and various metabolic factors. Utilizing size exclusion chromatography followed by ultrafiltration, EVs were extracted from 2 mL of actively secreted saliva. EVs were characterized using nanoparticle tracking analyses, Western blot, and scanning transmission electron microscopy. Finally, group comparisons and bivariate correlation analyses were conducted. Results: Among the total cohort, the median size of salivary EVs was 190.05 nm, and the overall concentration ranged from 1.4 × 10^7^ to 1.76 × 10^9^ per mL of saliva. The size range and concentration of EVs per mL are negatively correlated (*p* = 0.0002, r = −0.264). Comparing lean participants (mean rank of 45.98) with those presenting obesity (mean rank of 34.46), a significant difference in the salivary EV content was observed (*p* = 0.029). Body weight, BMI, arm and calf circumferences, as well as the percentage of body fat were all negatively related to the concentration of EVs in all study participants (all *p* < 0.05, r > −0.2). No associations were found between the EV parameters and taste perception but serum alkaline phosphatase levels were negatively correlated (*p* = 0.007, r = −0.284) and adiponectin serum levels were positively correlated to the EV concentration (*p* = 0.036, r = 0.208). Conclusion: The current study provides evidence for the relation between salivary EVs and anthropometric as well as metabolic parameters of obesity. This can provide the basis for further research on the cargo of salivary EVs and how they may influence taste sensation, and may elucidate their potential connection to altered eating habits in obesity.

## 1. Introduction

Extracellular vesicles (EVs), membrane-bound particles that are secreted from all body tissues, play a pivotal role in inter-organ communication by transporting genomic information and signaling molecules such as proteins, lipids, and nucleic acids, to recipient cells enabling various biological functions [1,2,3]. Based on their size and biogenesis, EVs can be categorized into several subtypes, the most notable being exosomes (30–120 nm) and microvesicles (150–1000 nm) [4]. Increasing evidence highlights the potential of EVs as biomarkers for various conditions. This refers mostly to circulating EVs shown to be increased in relation to malignant, inflammatory, and neurological diseases, but also in relation to metabolic conditions such as cardiovascular disease, type 2 diabetes mellitus, and obesity [5,6,7,8]. Obesity has thereby been linked to an overall increased number of circulating exosomes and adipose tissue EV-derived microRNAs are differentially expressed compared to normal weight [9,10]. These adipocyte-derived EVs (adEVs) contribute to insulin resistance by affecting pancreatic islet inflammation along with changes in insulin secretion, and changes in the hepatic mRNA expression have been linked to noncoding RNAs derived from adEVs [11,12,13]. By affecting proopiomelanocortin neurons, adEVs target hypothalamic anorexigenic pathways, and mice show altered eating behavior as a consequence of intravenously injected exosomes derived from visceral [14]. Administration of these adEVs from obese mice into the circulation of lean mice resulted in increased food intake. Additionally, daily foods contribute to the exosome load in humans with an impact on overall health [15]. Mice fed a high-fat diet present significant alterations in their circulating EV composition with effects on whole-body metabolism, while this type of diet greatly affects EV biogenesis, their fat content, and how they interact with body tissues [16]. However, while a large body of evidence points towards circulating EVs as critical indicators of obesity and co-morbidities, large amounts of blood material are needed for studying the EV content. This makes blood not the ideal source for EV biomarker detection and other biomaterial such as saliva might be beneficial. Indeed, saliva harbors substantial amounts of EVs, is produced in quantities of 500–1500 mL per day, and its collection is non-invasive and inexpensive. In general, studies show a limited salivary flow rate, increased viscosity, different protein and metabolite profiles, as well as altered salivary bacteria composition under obese conditions [17,18,19,20,21]. These differences have been related to flavor perception, underscoring the relevance of saliva when investigating obesity and eating behavior [22]. Moreover, people suffering from obesity, including children, display an abnormal taste sensitivity as well as different processing of certain flavors and odors compared to normal-weight individuals [23,24,25,26,27,28]. Changes in salivary adipokine levels under obese conditions have been demonstrated and are known to affect taste cell signaling by directly interfering with their receptors on taste bud cells [29,30,31,32]. Although local cells including taste cells are of an endocrine nature, salivary EVs may also contribute to total hormone levels in saliva. Recent studies have demonstrated the potential of an adoptive transfer of adiponectin-enriched EVs in mitigating weight gain in mice fed a high-fat diet [33]. This indicates that EVs act as conveyors of adipokines, thereby modulating metabolic processes and potentially counteracting the effects of obesity [34]. Interestingly, adiponectin and leptin have been linked to EV production or their release from donor cells outside of the oral cavity [30]. Beyond hormones, EVs may also transfer other factors into the oral cavity, with effects on taste sensation and oral health in general. Indeed, like circulating EVs, salivary EVs and especially their non-coding RNA cargo have been related to a variety of oral and non-oral cancers [35,36]. They are also important for periodontal health, are related to Sjogren’s syndrome, and have been implicated in tissue repair, including taste bud regeneration upon tongue damage [37,38]. Salivary EVs may thereby be derived from local tissues such as the salivary glands or from taste bud cells, but may also derive via the bloodstream from distant tissues including adipose tissue, or originate from daily diet [39,40]. However, the question if salivary EV composition is affected by obesity has not been addressed so far. As several studies link diseases including obesity to increased numbers of circulating EVs, we hypothesize that the salivary EV content is increased upon obesity, which is related to taste ability [9]. Therefore, this study aimed to investigate whether the concentration and size distribution of salivary EVs differ between people with normal weight and obesity and whether these factors are related to anthropometric and metabolic parameters, including adipokines. Moreover, potential contributions of salivary EVs to taste ability and taste bud density (TBD) were addressed.

## 2. Materials and Methods

### 2.1. Study Design and Data Collection

For the present study, 119 participants of the Obese Taste Bud (OTB) Study were included [25]. Briefly, the OTB Study (NCT04633109; DRKS00022950) was initiated in 2020 at the University Hospital of Leipzig in collaboration with the HI-MAG Institute as a cross-sectional observational study aiming to address the effects of obesity on the sense of taste. The study was conducted in accordance with the Declaration of Helsinki, and the protocol was approved by the Ethics Committee of the University of Leipzig (011/20-ek, approval date: 19 March 2020). People with a body mass index (BMI) >18 and aged between 18 and 69 years were included after a telephone screening for eligibility and absence of contraindications such as severe kidney, heart, liver, neurological, or mental disease, including eating disorders, and diseases directly impacting taste perception. Further exclusion criteria were diagnosed malignant diseases, current or history of radiation therapy, chemotherapy, or recent surgery, as well as current substance abuse, steroid use, pregnancy, or breastfeeding. Data and biomaterial collection was performed on two days. After an overnight fasting period of >12 h, participants arrived at the outpatient clinic of the University Hospital or the HI-MAG facility at 7:30 a.m. and gave written informed consent before participating in the study. Data collection included a fasting blood draw, taste and smell tests, the assessment of anthropometric measures, a bioimpedance analysis, as well as saliva sampling and a taste bud density assessment, among others. Details on data assessment and the complete study protocol are described elsewhere [25].

### 2.2. Investigation of Study-Specific Outcome Variables

Whole saliva samples were collected from fasting participants who refrained from drinking, smoking, chewing gum, or brushing their teeth for at least 30 min prior to sampling. To ensure the absence of acute oral pathologies, a brief inspection of the oral cavity was conducted prior to sample collection. Adhering to the Minimal Information for the Study of Extracellular Vesicles (MISEV), saliva samples were collected without any stimulus through drooling into clean, sterile falcon tubes within a 5–7 min window, for obtaining approximately 2 × 2 mL of actively secreted saliva, one sample for EV preparation and one for analyzing other factors such as hormone levels [41]. The collected samples were kept on ice and processed within 1.5 h after sampling by either directly freezing or pre-processing through centrifugation at 3500× *g* for 20 min at 4 °C to remove debris and then stored at −80 °C. A fasted blood draw was conducted, and serum and plasma were either kept on ice before further preparation and analysis or analyzed at the Institute of Laboratory Medicine, Clinical Chemistry and Molecular Diagnostics (ILM), of the University of Leipzig Medical Center. Investigated parameters included serum glucose, insulin levels, and lipid levels, among others, as stated elsewhere [25]. Anthropometric data included body height (m) and weight (kg) obtained on a SECA stadiometer as well as the measurement of waist, hip, arm, and leg circumferences (all in cm) with consecutive calculation of BMI (kg/m^2^) and waist-to-hip ratio (WHR). Bioimpedance analyses (BIA) provided data on body composition, including the percentage of body fat. Taste ability of the five basic tastes, i.e., sweet, sour, salty, bitter, and umami, was assessed using commercially available taste strips (Burckhart ODOFIN taste strips, Medisense, Stuttgart, Germany) impregnated with saccharose, citric acid, natrium chloride, quinine hydrochloride-dihydrate, and glutamate, respectively. The taste strips were presented in a randomized order and in four increasing concentrations per taste by placing a single taste strip in the center of the participant’s tongue. After each trial, the participants rinsed their mouths with water without swallowing and evaluated the presented taste via LimeSurvey thereafter [42]. Taste ability for each taste quality was obtained by calculating the sum of correctly identified taste strips ranging from 0 to 4. A total taste score was defined ranging from 0 to 16 of correctly identified tastes, including all tastes except umami. Taste bud density (TBD) was obtained by applying commercially available blue food color on the participant’s tongue, allowing easy visualization of fungiform taste buds. After placing a reference filter paper with an area of 177 mm^2^ on one side of the tongue, a digital photograph was taken and analyzed following a standardized protocol to obtain the number of taste papillae within the reference area.

### 2.3. Isolation of Salivary EVs

EVs were prepared from the debris-free supernatant (approximately 2 mL) of centrifuged saliva samples from each participant by size exclusion chromatography (SEC) using the ÄKTA Purifier 10 system (GE Healthcare, Munich, Germany). A Cytiva Superdex™ 200 Increase 10/300 GL column with a pore size of 8.6 µm was employed and separation was performed in phosphate-buffered saline (PBS) with a controlled flow rate of 0.4 mL/min. Absorbance was measured at 220 and 280 nm to monitor the fractions being eluted. From each sample, all elution fractions between 10 and 600 kDa were collected and stored at −80 °C until further processing. For subsequent experiments, the isolated fractions were concentrated using Vivaspin^®^ 6 columns (SARTORIUS, Göttingen, Germany) with a 10 kDa cut-off and by centrifugation at speeds ranging from 1000 to 4000 rpm and for durations ranging from 5 to 20 min depending on the characteristics (e.g., particle density) of the sample. Isolated EVs were stored at −80 °C until further analyses.

### 2.4. Characterization of Isolated Salivary EVs

According to the MISEV criteria, several methods were applied to validate the presence and effective isolation of EVs from saliva. The complete protocol confirming the isolation of EVs is described in the Supplementary Materials.

#### 2.4.1. Nanoparticle Tracking Analysis (NTA)

To quantify isolated particles and assess their size distribution, NTA analyses were applied using the NanoSight LM10 equipped with the NTA 3.0 software (Malvern Panalytical, Kassel, Germany). Per sample, all elution fractions from the SEC were separately analyzed in sterile filtered phosphate-buffered saline (PBS) by injecting 1 mL into the instrument using a clean syringe. Data were obtained by calculating the mean value of readings from 3 videos with 60 s duration each using the light scatter mode and constant settings such as target temperature of 25 °C, screen gain of 10, and detection threshold of 4. Elution fractions per sample were only used and pooled for subsequent analyses if all particles were <1000 nm in size, which was set as the cut-off for the definition of extracellular vesicles in the present study. The final number of isolated EVs/mL/per sample was calculated as the mean value from all remaining elution fractions per sample. Size distribution was given for each sample by D10, D50, and D90 levels, indicating that 10%, 50%, or 90% of the sample volume contained particles lower than these values. The overall median size of particles was used for correlation analyses.

#### 2.4.2. Western Blot and Simple Western Blot WB/JESS

For further proof of EVs to be present in isolated sample fractions, a detection of EV markers using Western blot analysis was performed. Due to the limited amount of sample material available, only a few study samples were included in these analyses. The total protein concentration of isolated EV-containing fractions from SEC was determined using the Pierce Micro BCA Protein-Assay-Kit (Thermo Scientific™, Waltham, MA, USA) according to the manufacturer’s protocol. In accordance with the concentrations measured, 3–5 samples per participant and elution fraction were pooled for qualitative assessment of EVs and subsequently concentrated. Equal amounts of protein (30–50 μg) were loaded onto a 10% SDS-PAGE and transferred onto a nitrocellulose membrane using tank blotting. The membrane was blocked using 1% BSA in TBST and incubated with primary antibodies at 4 °C overnight. Primary antibodies included anti-Calnexin (610524) as a negative marker for intracellular content and the Extracellular Vesicle Human CD9/CD63/CD81 Antibody Panel (#100-0211, STEMCELL technologies, Cologne, Germany) to detect typical EV markers. Antibodies were applied at concentrations ranging from 1:1000 to 1:5000. The next day, the secondary antibodies anti-mouse IgG, HRP-linked (#7076), or anti-rabbit IgG, HRP-linked (#7074), were incubated for 2 h at concentrations ranging from 1:1000 to 1:20.000 (STEMCELL technologies, Cologne, Germany). For the detection of HRP conjugates on the immunoblot, the chemiluminescent substrate ECL (#W1001, Promega, Walldorf, Germany) was used. Equal volumes of Peroxide and Luminol Enhancer Solution were mixed shortly before use and then applied to the immunoblot. Evaluation of protein bands was conducted using the Genesys program V1.8.2.5 (Syngene, Cambridge, UK), with a preference focus of 76. Gene Tool software version 4.3.15.0 (Syngene, Cambridge, UK) facilitated further analysis. To optimize EV marker evaluation in the limited amount of starting material, the JESS simple Western system (BioTechne, Wiesbaden-Nordenstadt, Germany) was used according to the manufacturer’s recommendations. Briefly, EV samples were diluted to 2.0 mg/mL, of which 3 µL was pipetted according to the JESS template, centrifuged for 5 min at 2500 rpm at room temperature, and run on the JESS system (BioTechne, Wiesbaden-Nordenstadt, Germany). Primary antibodies anti-ALIX (A302-938A, ThermoFisher Scientific, Darmstadt, Germany), anti-TSG101 (A303-506A, ThermoFisher Scientific, Darmstadt, Germany), and anti-PPARg (EPR18516, Abcam, Cambridge, UK) were diluted with Antibody Diluent 2, and corresponding secondary antibodies were provided as HRP conjugates ready to use. The Compass Software version 6.0.0 (BioTechne, Wiesbaden-Nordenstadt, Germany) was used for both the experimental process and subsequent analysis for the detection of applied EV biomarkers.

### 2.5. ELISA

For the evaluation of serum and/or salivary adiponectin, leptin (#E09 and #E07, respectively; Mediagnost, Reutlingen, Germany) and insulin levels (#10-1132-01; Mercodia, Uppsala, Sweden) in samples from the study cohort, ELISA was applied following the manufacturer’s instructions. Briefly, samples of directly frozen saliva were thawed on ice and centrifuged at 1150× *g* for 15 min at 4 °C, and 100 µL of undiluted saliva supernatant was used for salivary adiponectin assessment. Salivary insulin was analyzed from 25 µL of 1:2 diluted saliva supernatants. Additionally, serum samples were analyzed for leptin and adiponectin using 20 µL of serum undiluted and 10 µL of serum diluted 1:310, respectively. Serum was obtained from blood samples kept on ice for 30 min to allow clotting prior to centrifugation at 3.500 rpm. Samples were aliquoted and stored at −80 °C until the ELISA measurements. Each experiment was performed in duplicate, with each experimental plate containing 2 non-template controls as well as a standard curve. Spectrophotometric analysis was conducted with a FlexStation 3 Plate reader (Molecular Devices, San Jose, CA, USA) at a wavelength of 450 nm to facilitate the quantification of the target biomolecules.

### 2.6. Statistics

Statistical analyses were conducted using SPSS statistics software version 29.0.2.0 (IBM, Böblingen, Germany) and GraphPad Prism Version 9.4.1 (GraphPad Software, Boston, MA, USA). Categorical variables were analyzed using proportions (%), while continuous variables with non-Gaussian distribution were expressed as medians with 25th and 75th percentiles. Normal distribution was checked using the Kolmogorov–Smirnoff test. Accordingly, group differences were assessed using the Mann–Whitney U test, and correlation analyses were computed using Spearman’s correlation coefficient for non-normally distributed data. Data were transformed using the natural logarithm (ln) prior analyses. Statistical significance was defined at a *p*-value of <0.05. Grubb’s test was performed to identify outliers, which were subsequently removed from association analyses, if applicable. An adjustment for the confounding variables BMI, age, sex, and smoking was performed, if applicable, and stated in the results section.

## 3. Results

### 3.1. Characteristics of the Study Cohort

For the examination of the abundance and size distribution of salivary EVs, 119 participants of the OTB Study were included, with 49 lean, 26 overweight, and 44 participants with obesity. Participants had a median age of 36 years; however, the lean subgroup was significantly younger, with a median age of 30 years compared to participants having obesity with a median age of 42 years (*p* = 0.006). The total cohort consisted of 62% females and 38% males and gender was evenly distributed between the lean and obese subgroups. As expected, significant group differences in lean versus participants with obesity were found regarding anthropometric and clinical parameters of the metabolic syndrome, which indicate a favorable metabolic profile in the lean subgroup (Table 1). Overweight participants were excluded for addressing group differences.

### 3.2. Characterization of Salivary EVs in the Total Cohort

EVs are of clinical interest because of their potential to shuttle information from one cell to a nearby or remote recipient cell. Despite recent evidence from adEVs to affect eating behavior, among other factors, knowledge about the diversity of salivary EVs related to obesity is scarce to date [14]. Therefore, we addressed this question by applying SEC and further downstream analyses to isolate salivary EVs from participants with and without obesity. A final number of 111 samples was included for subsequent data analyses after the exclusion of outliers.

**Figure 1:**
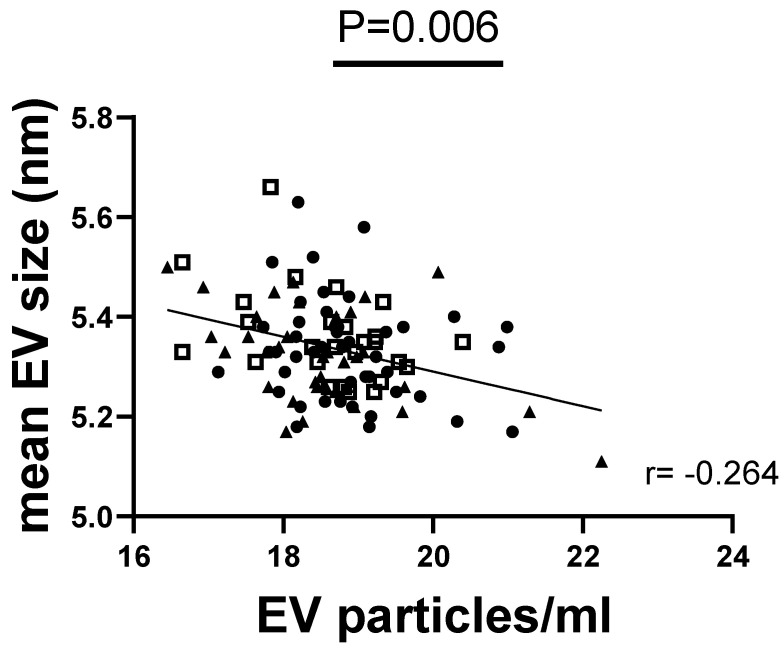
**Salivary EV size is correlated with particle concentration.** Spearmen correlation analysis between the median size of EVs and the particle concentration per mL in the total cohort (N = 111). A *p*-value < 0.05 was considered statistically significant. Lean participants are indicated as points, overweight as squares, and obese as triangles. EV = extracellular vesicles; *p* = *p*-value; r = correlation coefficient.

The results from NTA analyses showed that the median size of the EV particles in the overall cohort was 190.05 nm with a size range of D10 to D90 from 118.3 nm to 335.6 nm, showing that 10% of the sample volume contains particles below the D10 levels and 90% below the D90 levels. Overall, the concentration of EVs ranged from 1.4 × 10^7^ to 1.76 × 10^9^ per mL of saliva sample in the total cohort. A negative correlation between the EV size and the EV concentration (*p* = 0.0002) in the overall cohort was observed, as shown in Figure 1.

**Figure 2:**
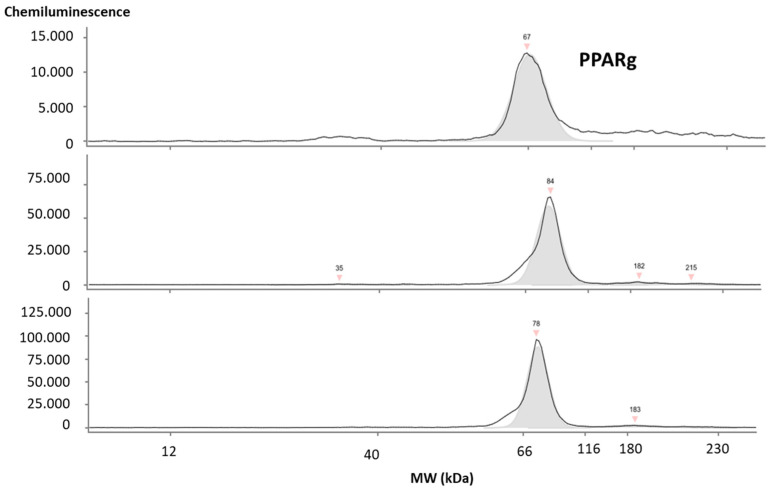
**Detection of PPARg in isolated salivary EVs.** PPARg was present in all 3 elution fractions obtained using the EV isolation protocol. All experiments were performed in 5 independent runs using separate patient samples. f1–f3 = elution fractions 1–3; EV = extracellular vesicles; MW = molecular weight; kDa = kilo Dalton; PPARg = peroxisome proliferating factor gamma.

Using scanning transmission electron microscopy (STEM) and Western blot, we confirmed the presence of EVs (Figure S2) and typical EV markers such as CD9, CD81, TSG101, ALIX, and the chaperones HSPA8/HSC70 and Flotilin-1 (Figures S3 and S4). We further demonstrated peroxisome proliferating factor gamma (PPARg) to be present within all elution fractions per sample, as shown in Figure 2.

Finally, by labeling the membrane of isolated EVs with PKH67 and subsequent application in HeLa cell culture, we demonstrated that salivary EVs were taken up by recipient cells and are thus likely to have functional consequences depending on their cargo (Figure S5).

### 3.3. The Concentration of Salivary EVs Differs between People with Obesity and Normal Weight

**Figure 3:**
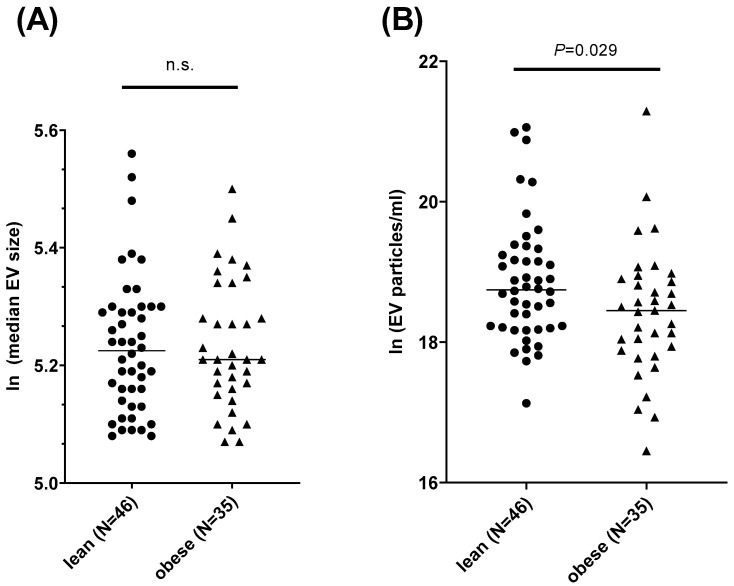
**Analysis of salivary EV size distribution and particle concentration in normal weight and people with obesity.** Group differences were analyzed using the Mann–Whitney U test and data are presented as median. (**A**) Median EV size: mean rank lean/obese = 40.61/41.51, U = 787, Z = −0.172. (**B**) Particle concentration: mean rank lean/obese = 45.98/34.46, U = 576, Z = −2.183. *p*-values < 0.05 were considered statistically significant. EV = extracellular vesicles; ln = natural logarithm; N = number; n.s. = not significant; *p* = *p*-value.

Increased levels of circulating EVs have been related to obesity, while salivary EVs have been mostly associated with malignant diseases so far [5,43]. Here, we addressed whether obesity parameters are related to the size and content of EVs. Since we could not identify significant correlations with age, gender, or current cigarette smoking, no adjustment for these potential confounders was applied. The median size of EVs did not differ between the groups (*p* > 0.05), with median particle sizes of 188.79 nm ± 334.97 nm in the obese subgroup and 188.56 nm ± 334.56 nm in the lean subgroup (Figure 3A). Lean participants tend to have a broader spectrum of EV sizes as compared to obese participants, as indicated by a higher standard deviation of the D10 levels obtained in the NTA measurement, meaning that 10% of the sample volume contained particles sized below 128 nm ± 134 nm in lean participants as compared to 109 nm ± 15.5 nm in the obese group. Regarding the concentration of EV particles, we found significant (*p* = 0.029) differences between lean participants and participants with obesity. However, the latter group yielded fewer particles per mL (Figure 3B).

### 3.4. The Concentration of Salivary EVs Is Correlated with Anthropometric Phenotypes

**Figure 4:**
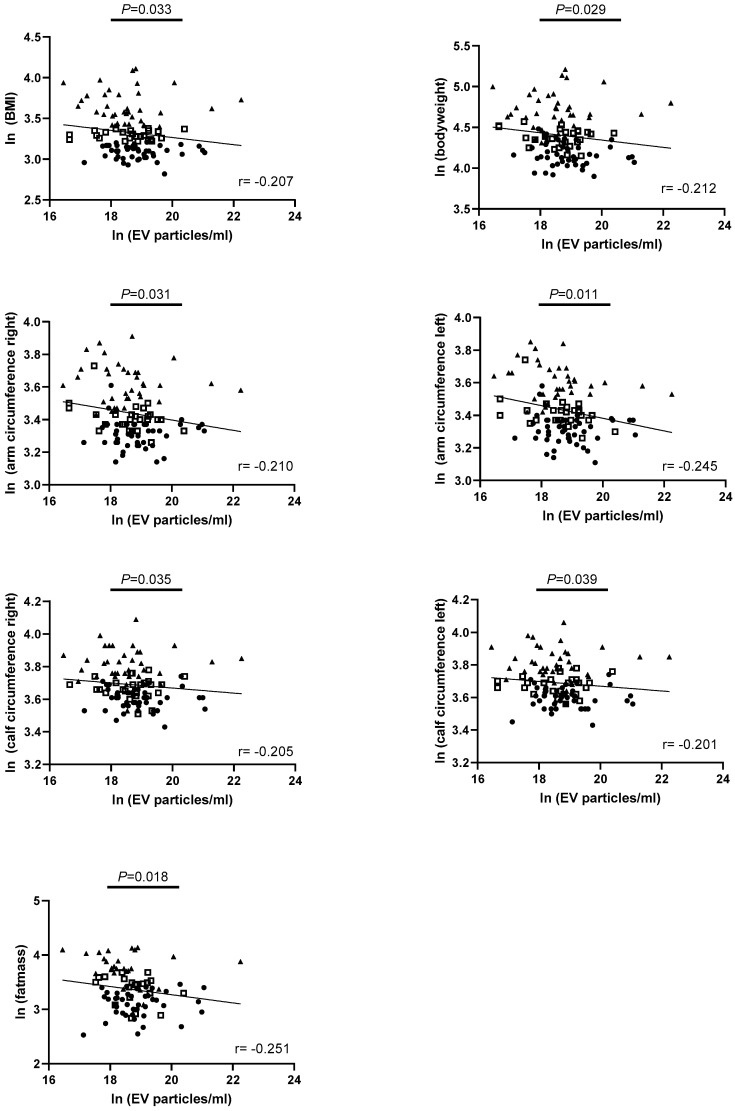
Correlation analyses between the salivary EV concentration and anthropometric parameters of obesity within the total cohort. Spearman correlation coefficient was used and *p*-values < 0.05 were considered statistically significant. Lean participants are indicated as points, overweight as squares, and obese as triangles. ln = natural logarithm; *p* = *p*-value; r = correlation coefficient.

Next, we performed correlation analyses to address the relationship between the size distribution and concentration of EVs within saliva samples of the total cohort and parameters clinically relevant to characterize obesity and associated comorbidities. We identified negative correlations with anthropometric parameters addressing the EV concentration in the overall study sample, as shown in Figure 4.

### 3.5. Exploring Correlations of EV Parameters with Metabolic Factors of Obesity, Taste Ability, and Lingual Taste Bud Density

**Figure 5:**
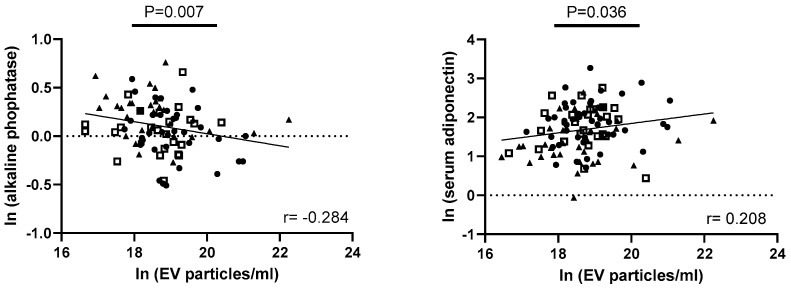
Correlation analyses between the salivary EV concentration and metabolic parameters of obesity within the total cohort. Spearman correlation coefficient was used, and *p*-values < 0.05 were considered statistically significant. Lean participants are indicated as points, overweight as squares, and obese as triangles. ln = natural logarithm; *p* = *p*-value; r = correlation coefficient.

We further investigated a potential link between serum levels of obesity-associated metabolic markers and EV size and concentration. A negative association was found between the EV concentration and the levels of alkaline phosphatase (ALK) in serum (*p* = 0.007), while a positive correlation was detected between the EV concentration and serum adiponectin levels (*p* = 0.036; Figure 5). These correlations did not withstand an adjustment for BMI. Also, no correlations were found with serum insulin or leptin levels.

The adipokines adiponectin, leptin, and insulin have been previously linked to EV biogenesis or release in extra-oral tissues to modulate taste perception or to affect taste bud density [30,31,32,44,45,46]. Therefore, we explored a potential link of salivary EVs to those factors. We could not identify a correlation of the EV concentration or the median size of EVs with the taste ability in the overall cohort, nor a correlation of these parameters with TBD. In the case of insulin and leptin (serum or saliva) levels, no correlation to taste abilities or TBD was found. However, we did find TBD to be moderately related to the sum score of taste ability (*p* = 0.048; r = 0.191) and adiponectin serum levels to be correlated with the ability to detect umami (*p* = 0.008; r = 0.248) and bitter (*p* = 0.032; r = 0.202).

## 4. Discussion

Previous studies have demonstrated that individuals with obesity display an overall impaired oral health status. This is accompanied by changes in the salivary flow rate, increased viscosity, and altered salivary protein composition, including inflammatory markers such as C-reactive protein, which are elevated in obese individuals [19,47,48]. Additionally, imbalances of salivary hormones, such as higher levels of insulin and leptin, have been associated with obesity in children, while these hormones, among others, are also involved in taste sensation [48,49]. Moreover, obesity has been linked to an altered taste bud transcriptome in humans, as well as a reduced fungiform papillae density [50,51] Furthermore, alterations in taste sensation have been discovered in relation to obesity and weight loss [50,51,52,53,54,55]. However, the mechanisms of these relationships are poorly understood. EVs might hold a potential solution, since these particles are involved in cell-to-cell communication by transferring genomic information even between distant tissues and cells [56]. Therefore, the aim of the present study was to characterize the EVs in saliva samples with regard to their size distribution and particle concentration, and to link these factors to parameters clinically relevant for obesity and taste sensation. We hypothesized the concentration of salivary EVs to be increased upon obesity, as shown before for circulating EVs [5]. Surprisingly, our results demonstrate a negative relationship with anthropometric parameters such as body weight, BMI, and the percentage of fat mass, which were identified as being inversely associated to the EV concentration in saliva. As obesity is linked to a reduced salivary flow rate and a more viscous saliva, it may be speculated that this negatively impacts the concentration of EVs in whole-mouth saliva [5]. However, data collected in the present study do not allow us to draw conclusions regarding the origin of salivary EVs. EVs might derive from cells of the salivary glands or other oral tissues, including taste cells, but they may also originate from the bloodstream or local bacteria. As the source of EVs is of high relevance regarding their cargo and functional consequences for target cells, future studies should include analyses of origin. The observation that PPARg was present in isolated salivary EVs might point towards an origin, at least for a part of these EVs, from fat depots or local adipocytes in the tongue muscle [57,58]. Interestingly, elevated tongue fat in obesity leads to, e.g., sleep apnea, but may also contribute to local inflammatory processes upon metabolic alterations in obesity with potential effects on the taste system [58]. On the contrary, ductal cells of the submandibular gland express PPARg, too, and thus, the origin of PPARg in salivary EVs is unclear [59]. In addition, we were only able to use a small number of samples for applying Western blot analysis since the sample material was limited. Hence, we cannot conclude that this transcription factor is present in the EVs of all study participants, and no information is available on the number of PPARg positive counts. Therefore, this finding needs to be interpreted with caution. Anyway, it would be highly interesting to replicate this current finding and to understand the functional consequence of this transcription factor in salivary EVs, since PPARg is not only the main driver of adipocyte differentiation, but is generally involved in stimulating lipid metabolism, hence nutrient signaling [60,61]. Moreover, in epithelial cells, including salivary duct cells, PPARg mediates anti-inflammatory effects and impaired functions contribute to Sjogren’s syndrome [59]. Therefore, it would be interesting to address the possibility that EV-transported PPARg mediates oral gene transcription of inflammatory or lipid-associated genes that may contribute to nutrient or taste signaling. This could enhance our understanding of obesity-associated alterations of taste signaling. So far, most studies identified salivary EVs to be relevant for oral cancers and systemic diseases mostly of malignant character [43,62,63]. Moreover, exosomes from oral tissue stem cells have been implicated in various biological processes, including immunomodulation, osteogenesis, neuroprotection, or vascularization, among others [64]. Alterations of salivary EVs in the context of obesity have not been investigated yet, in particular not in relation to taste perception in obesity. Such implications are likely, since mesenchymal stem cell-derived EVs injected into the oral cavity of a defective tongue mouse model were found to be involved in tissue repair, including taste bud regeneration [38]. Additionally, these exosomes were found to enhance the innervation of regenerated taste buds, indicating their potential in restoring taste function. Additionally, exosomes have been implicated in taste dysfunction following infection with COVID-19 [65].

It has been shown previously that adipokines are cargos of EVs while also being able to affect EV biogenesis and release. Hence, they may also contribute to the EV content of the oral cavity [30,44,45]. Especially adiponectin, derived from fat cells, is loaded into EVs and transported via the bloodstream towards the pancreas, mediating effects on ß-cell function with effects on insulin secretion [12,66]. On the contrary, adiponectin is able to stimulate the generation and release of exosomes in mesenchymal stem cells (MSC), which mediates the effect of MSC transplantation as a therapy for heart failure in mice [67]. Furthermore, patients with metabolic syndrome present with a lower amount of EVs with medium size, which was related to fasting insulin levels accompanied by consequences of insulin sensitivity, while studies show leptin to mediate the release of EVs from breast cancer cells [44,45]. Additionally, leptin, insulin, and adiponectin are related to food intake and are known to affect the perception of sweet, salty, and fat, respectively, by directly impacting taste cell signaling [30]. While leptin binds to its receptor in sweet-sensing taste cells to modulate local K^+^_ATP_ channels and thus blunt the sweet response, insulin can reduce responses to NaCl signaling [31,32,68], and adiponectin has been connected with functional relevance in the regulation of the fat receptor CD36 and consequent fat consumption in mice [46]. In the current analyses, we could not observe associations between insulin and leptin levels with taste ability. It might be of future relevance to evaluate a potential oral tissue resistance in obesity, which could blunt the effects of insulin and leptin signaling and may partially explain the controversial findings from the current analyses. However, we observed a relationship between serum adiponectin levels and the ability to detect umami and bitterness. Previously, adiponectin was not related to taste sensation of umami or other flavors except for dietary fat [69]. Here, we have not addressed fat taste perception and the described discrepancies may arise from other taste substances being employed, diversity in sample sizes, or various other factors, including study design or the time of the day for data assessment. Moreover, species-specific effects may play a role, since previous studies on adiponectin affecting taste sensation were performed in mice, while in the current analyses, a human dataset was employed. Nevertheless, we identified salivary EVs being increased with higher serum adiponectin concentrations. It may be speculated that systemic adiponectin mediates the release of EVs into the circulation, which are finally transported through the endothelial barrier into the oral cavity and hence can contribute to local EV content. Studies demonstrated a clear link for extra-oral EVs impacting TBD upon tissue repair, as mentioned before. Although we saw TBD being linked to the overall taste ability, no involvement of salivary EVs in both factors was found in the data analyses. On the contrary, the potential of EVs to affect taste signaling, has recently been shown [32]. The activation of the TAS2R14 bitter receptor in JAR cells by its agonist diphenhydramine resulted in an increased release of EVs with effects on intracellular calcium levels [70]. Interestingly, fatty acids, among several other metabolic factors, have been shown to be involved in the sorting process of the vesicle cargo and the type of vesicles being generated [71]. In the context of missing links between EV concentration and taste signaling, it might be of potential interest to study specific salivary EV types, their origin, and cargo, rather than the focus on the total EV load. We believe that metabolic alterations arising with obesity affect the taste perception and signaling pathway potentially by influencing the load and cargo of salivary EVs. Therefore, we tested a row of metabolic factors specifically related to obesity as the primary outcome of this manuscript. However, in general, metabolic alterations may influence the salivary EV composition. Therefore, we also tested a row of other factors and, interestingly, we found ALP, which is a highly sensitive marker for liver or bone metabolism, to be associated with the salivary EV concentration. However, further work is needed to understand the contribution of metabolic alterations on salivary EVs in general. In summary, we demonstrated a negative relationship between salivary EV concentration and obesity. If and how this contributes to taste dysfunction in obesity will be the subject of future studies.

### Strength and Limitations

The current study is limited by several aspects. First, the cohort is rather small, limiting the statistical power to detect effects, and the results obtained need to be interpreted with caution to avoid overestimation. This is indicated by the results obtained by Spearman’s rank tests as correlation coefficients between −0.3 and +0.3 are interpreted as weak effects. Although we applied an adjustment for confounders such as BMI, age, sex, and smoking, we cannot rule out the potential for further confounding. Furthermore, only exploratory descriptive analyses were applied in the current work, hindering us from drawing any functional conclusions. A selection bias could have influenced the results presented in this study, as the study was advertised at the campus of the University Hospital of Leipzig. This resulted in the enrollment of many students, especially in the control group, which is therefore rather young, highly educated, and likely more interested in a healthy lifestyle. On the contrary, the study includes participants with a high range of BMIs due to the study facilities being close to the obesity treatment center of the University Clinic Leipzig, which enabled us to specifically advertise the study to patients with obesity. Beyond that, only people living in Germany were included in this study. Studies on taste perception and food preferences are very heterogeneous due to a large variety of potential taste molecules that can be investigated and various questionnaires can be applied to address these phenotypes. More detailed analyses, including more complex taste flavors, may clarify the current discrepancies. A very strict protocol was followed for data acquisition, especially regarding the time frame of saliva collection. This was to avoid the impact of diurnal rhythms of protein secretion potentially interfering with EV release. Studies focusing on several time points of data assessment would be of relevance to prove the observed effects over the course of a day. Regarding the characterization of salivary EVs, it should be noted that the applied NTA is not suitable to discriminate between the particles being analyzed. Consequently, all particles in the solution are counted as EVs, and no pull-down analyses such as fluorescence-activated cell sorting (FACS) have been applied to overcome this issue. However, the isolation protocol was strictly evaluated and isolated EVs have been thoroughly characterized. Moreover, this study shows the efficient isolation of EVs from saliva in a very limited amount of starting material. This will help to strengthen the use of EV analyses in diagnostic approaches. Although the sample set is small, the dataset is rare and several omics levels will be available from the OTB cohort, such as transcriptomics from taste bud biopsies but also RNA sequencing data from the salivary EVs. Hence, follow-up studies will allow a more comprehensive data analysis. A focus on the EV cargo will thereby strengthen the understanding of functional effects on taste cells that may derive from salivary EVs.

## 5. Conclusions

The current research has yielded new insights into the role of salivary EVs in the context of obesity as a systemic, non-malignant disease. Although the data presented here are of an exploratory nature, a link between the salivary EV concentration and obesity was observed, including anthropometric data and serum adiponectin levels. Future work is needed to address the functional consequences of reduced salivary EV contents in obesity, with a focus on vesicle cargo.

## Figures and Tables

**Table 1:** Characteristics of the study cohort.

Clinical Parameter	N	Total Cohort	N	Lean	N	Obese	*p*-Value
**sex (% female/male)**	119	62/38	49	64/37	44	66/34	0.791
**age (years)**	119	36 (26–49)	49	30 (24–45)	44	42 (30–54)	**0.006**
**height (cm)**	119	1.72 (1.64–1.77)	49	1.73 (1.65–1.78)	44	1.71 (1.63–1.75)	0.389
**weight (kg)**	119	80 (65–101.8)	49	63.2 (58.25–74.15)	44	106.05 (91.73–124.5)	**<0.001**
**BMI (kg/m²)**	119	26.66 (22.52–34.01)	49	22.26 (20.62–23.53)	44	35.87 (31.73–44.11)	**<0.001**
**waist (cm)**	119	89 (75–104)	49	74 (70.5–81)	44	112.5 (100.38–123.5)	**<0.001**
**hip (cm)**	119	102 (91.5–114)	49	91 (87–95.5)	44	122 (109.88–135)	**<0.001**
**WHR**	119	0.86 (0.8–0.93)	49	0.82 (0.77–0.87)	44	0.91 (0.84–0.96)	**<0.001**
**fat mass (%)**	98	29.5 (21.95–40.4)	45	23.83 (18.94–27.18)	33	43.10 (39.79–54.45)	**<0.001**
**systolic BP (mmHg)**	70	120 (111–130.5)	21	112 (105–120.5)	28	125.5 (117–139.75)	**0.004**
**diastolic BP (mmHg)**	70	78 (73–84.25)	21	75 (68.5–81)	28	80 (76–88.5)	**0.008**
**ALP (µkat/L)**	100	1.13 (0.95–1.35)	40	1.04 (0.90–1.24)	36	1.32 (1.07–1.49)	**<0.001**
**total cholesterol (mmol/L)**	102	4.75 (4.2–5.58)	41	4.56 (4.02–5.19)	37	4.81 (4.31–5.49)	0.152
**non-HDL cholesterol (mmol/L)**	117	3.2 (2.48–3.94)	48	2.77 (2.26–3.54)	43	3.52 (2.8–4.03)	**<0.001**
**HDL cholesterol (mmol/L)**	102	2.89 (2.29–3.71)	41	2.6 (1.98–3.18)	37	3.1 (2.64–3.71)	**0.013**
**LDL cholesterol (mmol/L)**	102	1.5 (1.25–1.93)	41	1.81 (1.49–2.14)	37	1.3 (1.06–1.51)	**<0.001**
**triglycerides (mmol/L)**	102	1 (0.76–1.57)	41	0.77 (0.66–1)	37	1.28 (0.98–2.01)	**<0.001**
**FFA (mmol/L)**	102	0.61 (0.45–0.78)	41	0.61 (0.47–0.78)	37	0.66 (0.55–0.82)	0.37
**HbA1c (%)**	114	5.4 (5.2–5.8)	45	5.3 (5.1–5.7)	43	5.6 (5.2–6.1)	**0.011**
**fasting glucose (mmol/L)**	116	4.74 (4.44–5.13)	47	4.46 (4.28–4.7)	43	5.12 (4.79–5.68)	**<0.001**
**fasting insulin serum (pmol/L)**	100	38.4 (28.15–82.4)	41	28.3 (22.7–38.9)	36	88.1 (69.95–125.68)	**<0.001**
**insulin saliva (pmol/L)**	107	10.25 (4.32–19.8)	48	7.4 (3.21–11.31)	36	18.87 (9.24–30.05)	**<0.001**
**adiponectin serum (µg/mL)**	114	5.33 (3.47–8.21)	47	6.75 (4.48–9.7)	42	3.91 (2.62–5.88)	**<0.001**
**adiponectin saliva (ng/mL)**	104	6.4 (2.53–16.68)	47	6.0 (2.20–17.10)	34	6.15 (2.80–15.92)	0.886
**leptin serum (ng/mL)**	110	11.6 (2.87–21.95)	46	3.84 (1.46–11.82)	40	21.58 (11.93–40.66)	**<0.001**

Data are presented as median (25th–75th percentile), except sex, which is given as proportion in %. Group differences (lean versus obese) were analyzed using Mann–Whitney U tests and *p*-values < 0.05 were considered statistically significant and are highlighted in bold. Missing phenotypes are a consequence of unsuccessful blood draws or hemolytic blood material, which was not taken forward for analyses. Blood pressure was only assessed in a subset of participants due to technical reasons. Body fat percentage was obtained using bioimpedance analysis which could not be applied to all participants due to test-specific exclusion criteria (e.g., metal implants). ALP = alkaline phosphatase; BMI = body mass index; BP = blood pressure; FFA = free fatty acids; HbA1c = glycated hemoglobin; HDL = high-density lipoprotein; LDL = low-density lipoprotein; N = number; WHR = waist-to-hip ratio.

## Data Availability

The original contributions presented in the study are included in the article/Supplementary Materials, further inquiries can be directed to the corresponding authors.

## Supplementary Materials

The following supporting information can be downloaded at: https://www.mdpi.com/article/10.3390/nu16162633/s1, Supplementary Figure S1. Nanoparticle tracking analyses of separated elution fractions by SEC. Three elution fractions (f1–f3) of one saliva sample obtained using SEC (size exclusion chromatography) are presented. The x-axis represents the average size/concentration and red error bars indicate ±1 standard error of the mean. Supplementary Figure S2. Scanning Transmission Electron Microscopy of salivary elution fractions containing EVs. All elution fractions obtained from size exclusion chromatography (f1–f3) of two independent samples are shown. Black arrows indicate examples of single EVs with a typical round shape and membrane embedded. The overall size range among all fractions was between 176 nm and 625 nm (analyzed with ImageJ version 1.54j). Scale bar = 1 µm. Grey dots and black crumbs are residues of the preparation procedure of the samples. Supplementary Figure S3. Detection of EV markers. Western blot analyses of 3 elution fractions obtained by size exclusion chromatography from two independent samples are presented. All fractions contained EVs as indicated by positive staining for CD9 and CD81 as classical EV markers, while showing negativity for Calnexin, a mitochondrial protein, indicating no intracellular components being present in the samples. All experiments were done in 5 independent replicates using separate patient samples. f1–f3 = elution fractions 1–3; EV = extracellular vesicles; M = mitochondrial protein (used as positive control for Calnexin); CD9 = tetraspanin 9; CD81 = tetraspanin 81; kDa = kilo Dalton. Supplementary Figure S4. Detection of Biomarkers of EV with JESS: The figure presents the results from Jess analysis for the detection of EV biomarkers. Panels A through E show the presence of specific protein markers across the three fractions. Each panel represents the detection of a different biomarker: (A) CD9, (B) CD81, (C) ALIX, (D) Flotillin-1, and (E) HSPA8/HSC70. The protein bands for each biomarker are present in all three fractions, indicating the consistent expression of these EV markers across the samples analyzed. Supplementary Figure S5. Cellular uptake of isolated salivary EVs by HeLa cells. (A) A bright field image of HeLa cells, (B) PKH67 labeled EVs (green), (C) DAPI staining of cell nuclei (blue), (D) overlay of PKH67 and DAPI staining, providing a detailed view of EV localization within HeLa cells. Scale bar = 20 µm.
