# Supplementary material for: The Concentration of Salivary Extracellular Vesicles Is Related to Obesity
Source: Nutrients. 2024 Aug 9;16(16):2633. doi: 10.3390/nu16162633 (PMC11356876; doi:10.3390/nu16162633)
Supplement: Supplementary file 1 [file nutrients-16-02633-s001.zip › nutrients-3115987-supplementary.pdf]

The concentration of salivary extracellular vesicles is related to obesity

Supplementary Material - Confirmation of isolated EVs from saliva

Size exclusion chromatography with the ÄKTA system allows a clear separation of salivary EVs

Following the MISEV criteria, several methods were applied to ensure targeted analyses of salivary derived EVs. Briefly, the isolation of EVs from saliva supernatant free of debris was done using a size exclusion chromatography with the ÄKTA system. After elution of the EV containing saliva fractions, nanoparticle tracking analyses (NTA) were performed for each separated elution fraction per sample to quantify particle concentration, aiming to understand extracellular vesicle abundance, and to assess the distribution of particle size, thus offering insights into their heterogeneity and potential functional diversity. Figure S1 shows an example of three collected elution fraction of one sample, with the typical size range of extracellular vesicles between 50 nm and 500 nm as determined by NTA analyses. As a result of some overlaps between the elution fractions obtained by SEC, all fractions contain particles of the same sizes as seen in the NTA results, confirming an optimal output of EVs.

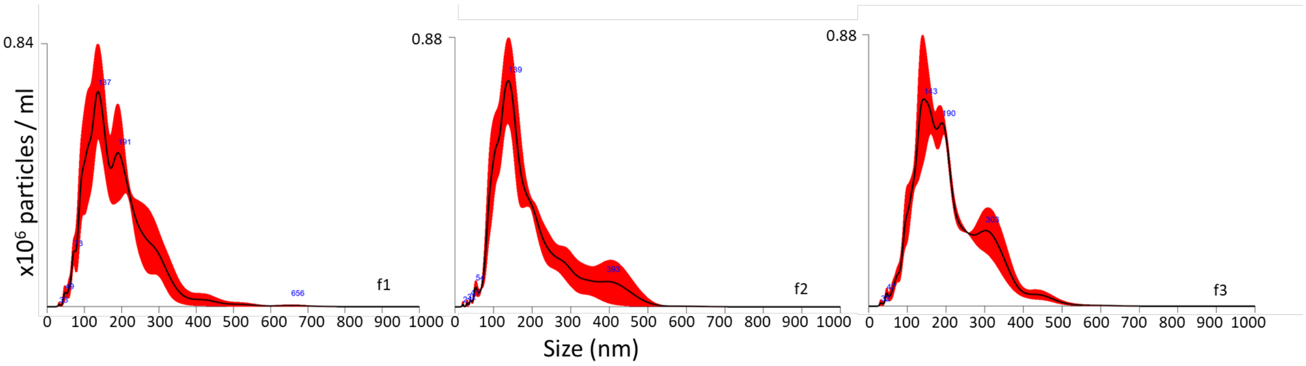

Supplementary Figure S1. Nanoparticle tracking analyses of separated elution fractions by SEC. Three elution fractions (f1-f3) of one saliva sample obtained using SEC (size exclusion chromatography) are presented. The x- axis represents the average size / concentration and red error bars indicate ± 1 standard error of the mean.

We further confirmed the presence of isolated EVs in all SEC elution fractions of the saliva supernatants by applying scanning transmission electron microscopy (STEM) analyses for a subset of samples. The sample solution containing concentrated salivary EVs was diluted 1:2 and fixed with 2% glutaraldehyde (Serva, Heidelberg, Germany) for 2 hours. Next, 5µl of the solution was transferred onto formvar-coated copper grids. After 5 min, the solution was removed with filter paper and the grids were placed in an aqueous solution of 1% uranyl acetate (Serva, Heidelberg, Germany) for 2 min. The samples were briefly rinsed in water and air-dried. STEM images were acquired with a Zeiss SIGMA electron microscope (Zeiss, Oberkochen, Germany). The size of EVs was measured with ImageJ software (free download from imagej.net). As shown in Figure S2, the presence of typical round, membrane embedded structures could be confirmed with size ranges from 176 nm to 625 nm over all fractions.

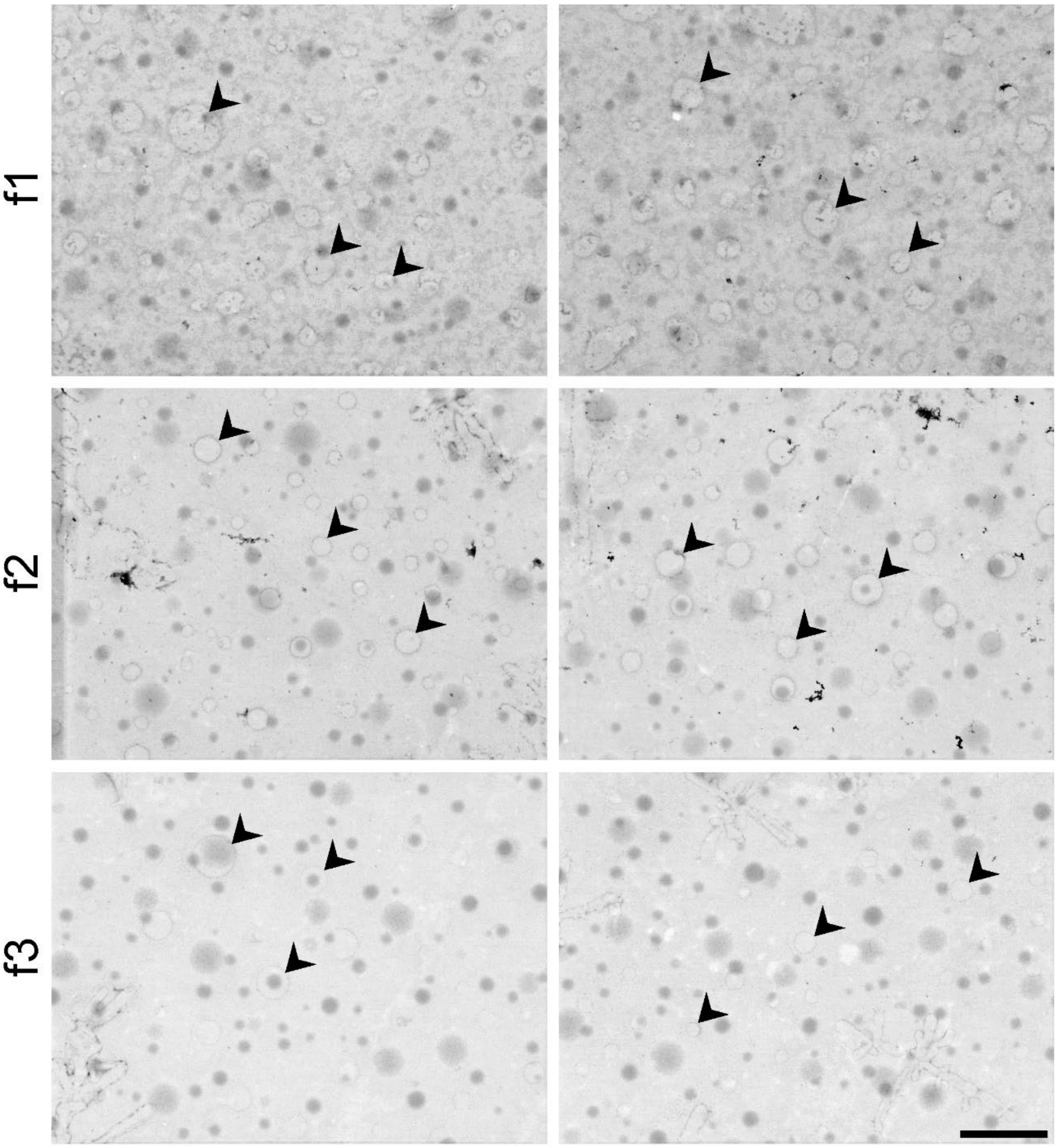

Supplementary Figure S2. Scanning Transmission Electron Microscopy of salivary elution fractions containing EVs. All elution fractions obtained from size exclusion chromatography (f1-f3) of two independent samples are shown. Black arrows indicate examples of single EVs with a typical round shape and membrane embedded. The overall size range among all fractions was between 176 nm and 625 nm (analyzed with ImageJ). Scale bar = 1µm. Grey dots and black crumbs are residues of the preparation procedure of the samples.

By western blot analyses, we confirmed the presence of classical EV markers such as CD9 and CD81 in each elution fraction per sample from a pool of 5 samples. The absence of calnexin, a protein present in the endoplasmic reticulum, provided evidence for a clear separation of EVs free of intracellular components. Data are shown in Figure S3.

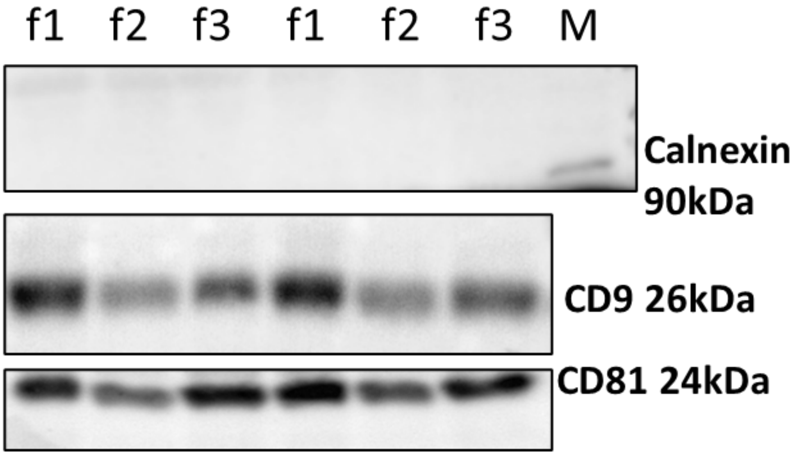

Supplementary Figure S3. Detection of EV markers. Western blot analyses of 3 elution fractions obtained by size exclusion chromatography from two independent samples are presented. All fractions contained EVs as indicated by positive staining for CD9 and CD81 as classical EV markers, while showing negativity for Calnexin, a mitochondrial protein, indicating no intracellular components being present in the samples. All experiments were done in 5 independent replicates using separate patient samples. f1-f3 = elution fractions 1-3; EV = extracellular vesicles; M = mitochondrial protein (used as positive control for Calnexin); CD9 = tetraspanin 9; CD81 = tetraspanin 81; kDa = kilo Dalton

Due to a high amount of starting material necessary for classical western blot analyses and the limitation of actual sample material, we additionally applied JESS (Just Enough Sample Stacking) as western blot method. The capillary-based automated western blot system primarily aims at enhancing sensitivity and accuracy in western blot analysis, by introducing notable advantages such as the reduction of required sample input to as low as 2 mg/ml and analyzing the samples without prior protein isolation, but directly loading EVs onto the analysis platform. With this, we could further confirm the clean isolation of EVs from saliva by showing the presence of CD9, CD81, TSG101, ALIX, as well as the presence of the chaperone HSPA8/HSC70 and the lipid raft-associated Flotilin-1 (Figure S4).

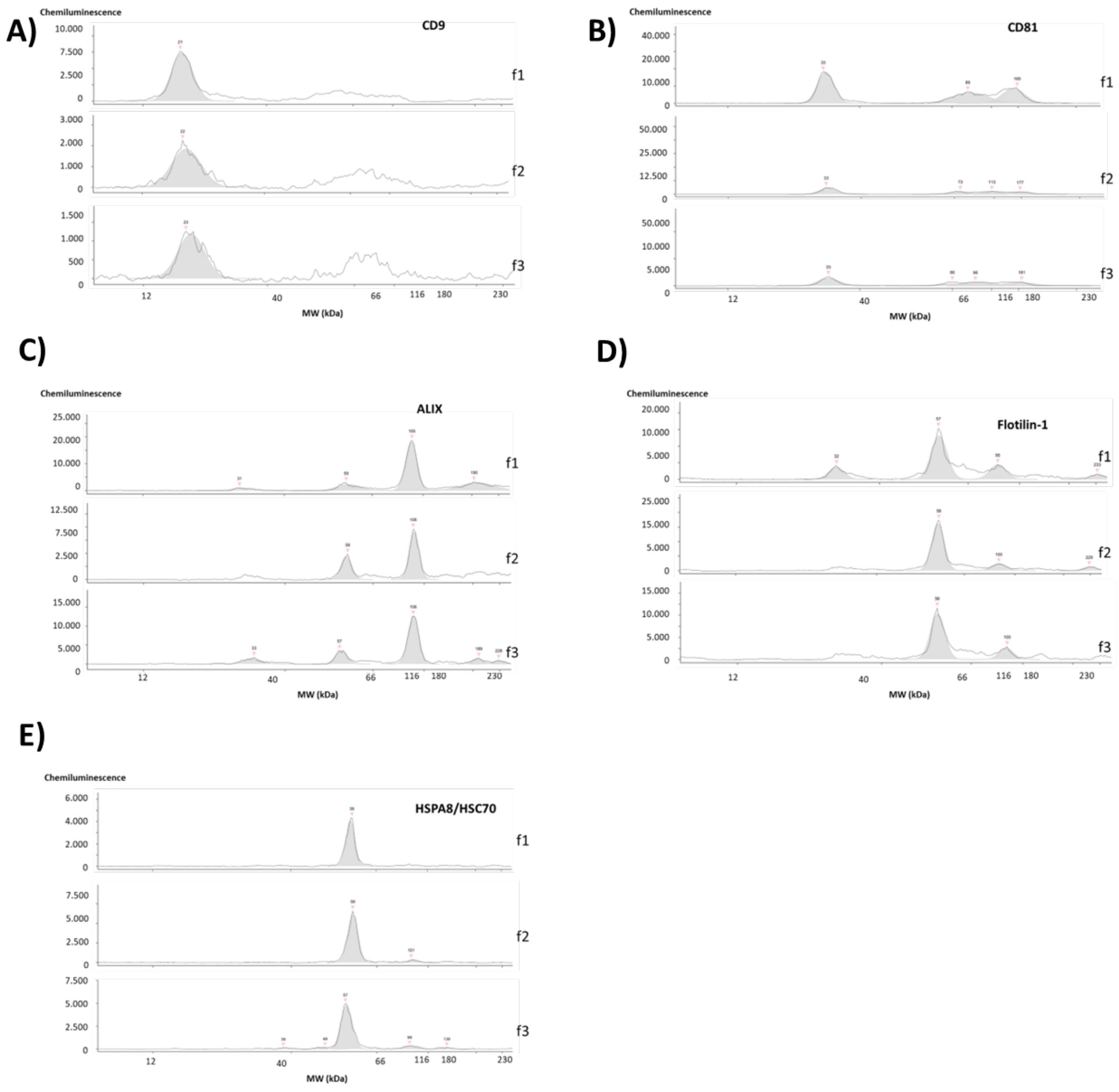

Supplementary Figure S4. **Detection of Biomarkers of EV with JESS:** The figure presents the results from Jess analysis for the detection of EV biomarkers. Panels A through E show the presence of specific protein markers across the three fractions. Each panel represents the detection of a different biomarker: A) CD9, B) CD81, C) ALIX, D) Flotillin-1, and E) HSPA8/HSC70. The protein bands for each biomarker are present in all three fractions, indicating the consistent expression of these EV markers across the samples analyzed.

Following the manufacturer's instructions, the PKH67 Green Fluorescent Cell Linker Mini Kit (Sigma Aldrich, Missouri, USA) was used to confirm the isolation of intact vesicles and EV functionality, by demonstrating EV uptake in HeLa cells. Isolated EVs from a validation sample were labeled with the green-fluorescent dye PKH67, a general membrane marker. Briefly, EVs were prepared according to the preparation of all other study samples, except for their concentration using ultracentrifugation at 100,000 x g. This was done to remove as much PBS as possible to avoid interference with subsequent procedures. For labeling, 25 µl of gently thawed EVs were mixed with 225 µl of Diluent C, prior to adding the sample to a mix of 1 µl PKH67 dye in 250 µl Diluent C. The suspension was incubated for 3 min at room temperature and the reaction was stopped by adding 1ml exosome free FBS, prepared by ultracentrifugation of FBS overnight at 100,000 x g.

HeLa cells were cultured in Dulbecco's modified Eagle's medium (DMEM) supplemented with 10% fetal bovine serum (FBS) and 1% Penicillin/Streptomycin. Two days before transfection, cells were seeded onto an 8-Well chamber slide (Ibidi, Gräfelfing, Germany) to allow cells to reach 70% confluency. One day before loading the cells with the salivary-derived EVs, the growth medium was replaced by medium containing exosome free FBS. Labeled EVs were resuspended in exosome-free cell culture medium. For transfection, medium in the Ibidi slides was replaced by 300µl of the labeled EV media mix and cells were cultured for 24 h, prior to monitoring. Cells were washed in PBS twice, fixed in 4% paraformaldehyde for 20 min and washed again in PBS. Excess PBS was removed and one drop of Ibidi mounting medium containing DAPI (Ibidi, Gräfelfing, Germany) was applied per well of the Ibidi slide for staining of cell nuclei. The Ibidi slide was analyzed using a Keyence fluorescence microscope at 500 nm for detecting DAPI and 490 nm for PKH67. As shown in Figure S5, EVs were taken up by the cells after 24 h indicating the isolation of intact extracellular vesicles.

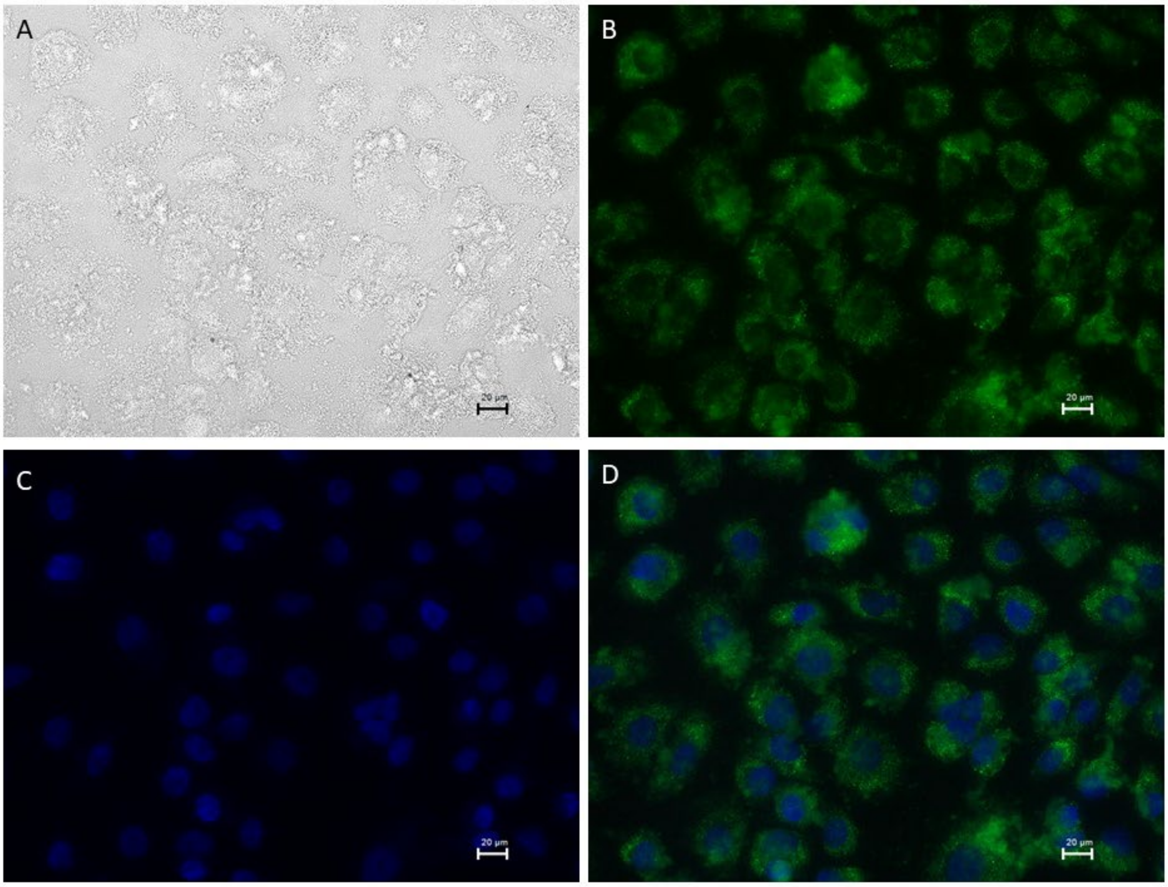

Supplementary Figure S5. Cellular uptake of isolated salivary EVs by HeLa cells. A) A bright field image of HeLa cells, B) PKH67 labeled EVs (green), C) DAPI staining of cell nuclei (blue), D) overlay of PKH67 and DAPI staining, providing a detailed view of EV localization within HeLa cells. Scale bar = 20µm.
